# A Virtuous Cycle of Phytoremediation, Pyrolysis, and Biochar Applications toward Safe PFAS Levels in Soil, Feed, and Food

**DOI:** 10.1021/acs.jafc.5c00651

**Published:** 2025-01-29

**Authors:** Gerard Cornelissen, Nathalie Briels, Thomas D. Bucheli, Nicolas Estoppey, Andrea Gredelj, Nikolas Hagemann, Sylvain Lerch, Simon Lotz, Daniel Rasse, Hans-Peter Schmidt, Erlend Sørmo, Hans Peter H. Arp

**Affiliations:** † Norwegian Geotechnical Institute (NGI), Oslo 0484, Norway; ‡ Norwegian University of Life Sciences (NMBU), Ås 1432, Norway; § ARCHE Consulting, Ghent 9032, Belgium; ∥ Environmental Analytics, Agroscope, Zürich 8046, Switzerland; ⊥ Ithaka Institute, Goldbach 63773, Germany; # Ruminant Nutrition and Emissions, Agroscope, Posieux 1725, Switzerland; 7) Norwegian Institute for Bioeconomy (NIBIO), Ås 1432, Norway; 8) Ithaka Institute, Arbaz 1974, Switzerland; 9) Norwegian University of Science and Technology (NTNU), Trondheim 7491, Norway

## PFAS in Agriculture

Farmlands can be contaminated with per- and polyfluorinated alkylated substances (PFAS) from increased levels in biosolids, compost, digestate, and animal manure. Such contamination can lead to high and persistent PFAS levels in (ground)water, crops, milk, and meat,^1^ increasing human dietary exposure.

## Phytoremediation, Pyrolysis, and Biochar Amendment

Remediation of PFAS-impacted agricultural soil is challenging because of the diffuse character of the pollution.^2^ Destructive approaches (soil washing, excavation, incineration, and chemical oxidation) will impair soil ecosystem services and cause carbon emissions.^2^*In situ* methods such as phytoremediation^3^ and sorbent amendment with carbonaceous and/or ion-exchanging materials^4^ are less intrusive and more cost-effective.^2,3^ Phytoremediation of PFAS has been demonstrated to be a cost-effective, environmentally friendly, energy efficient, and aesthetically pleasing option.^3^ However, high variabilities were observed between the uptake potential of different PFAS and between plant species.^3,5^ Pyrolysis can mineralize the PFAS in the phytoremediation biomass,^6^ providing a win–win solution in which PFAS is eliminated from biomass^6^ and other biosolids^7^ through pyrolysis, generating biochar. This is a sustainable sorbent material^4,8^ with co-benefits in terms of carbon sequestration (1–2 t of CO_2_ equivalents/t of biochar^9^), sustainable waste management,^2,6^ and energy generation during pyrolysis.^2^

## A Virtuous Cycle

**Figure 1:**
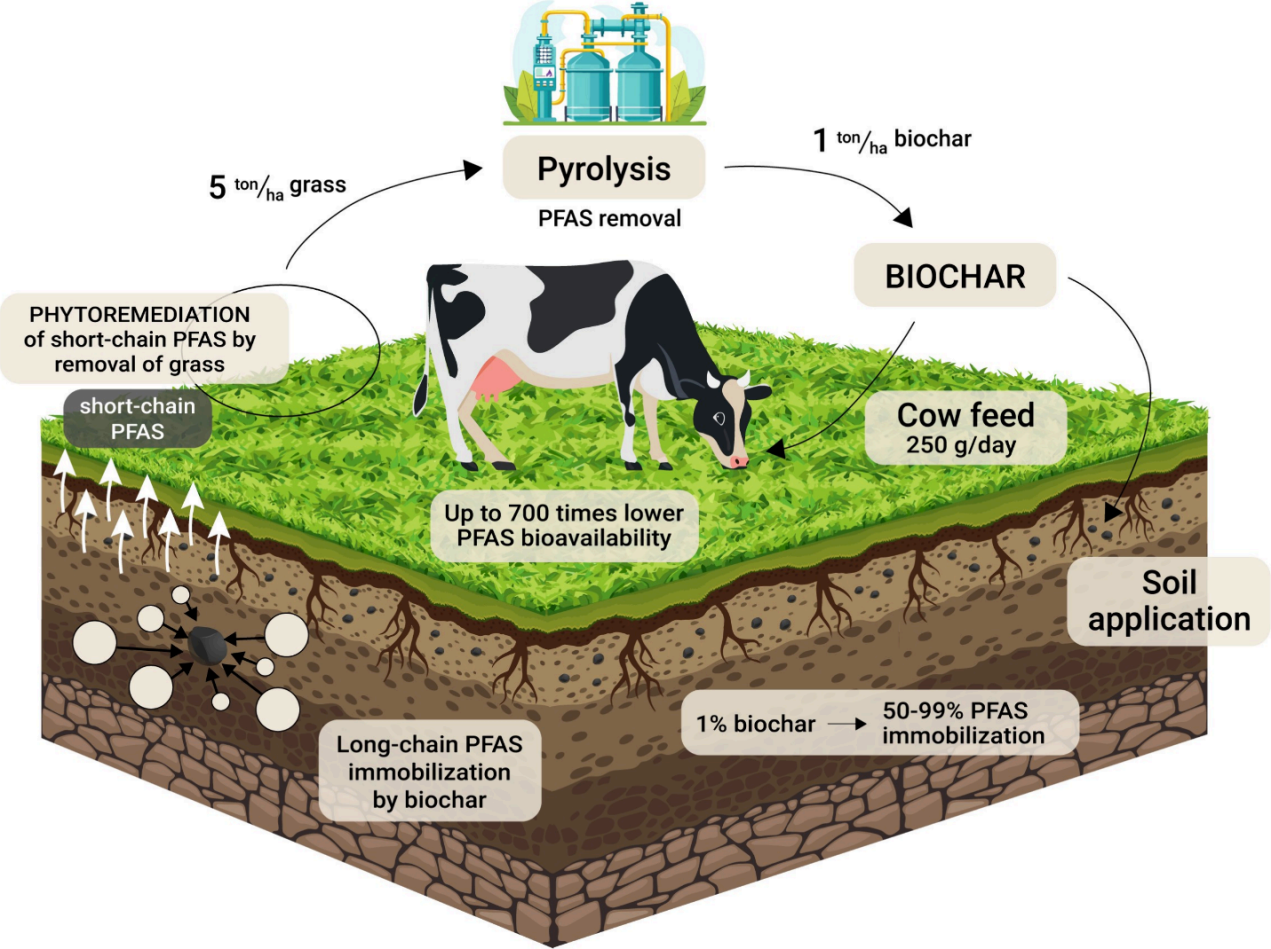
Phytoremediation–pyrolysis–biochar virtuous cycle including biochar-amended soil and ruminant feed.

We propose a virtuous cycle by using phytoremediation for the accumulation of short-chain PFAS, destroying them by pyrolytic treatment, and applying the resulting PFAS-free biochar as a sorbent to immobilize long-chain PFAS (Figure 1). Pyrolyzing the contaminated plant biomass alleviates the constraints of biomass disposal. The proposed cycle takes advantage of the high phytoextraction potential for (ultra)short-chain PFAS, which are less strongly sorbed to biochar. We further suggest that the addition of biochar to forages may reduce the uptake and bioavailability of PFAS, thereby reducing PFAS contamination in milk and meat.

To optimize the combined remediation by this cycle, pyrolysis probably needs to be conducted above 800 °C to ensure PFAS destruction^6^ and sufficient size of the pores in the biochar (>2 nm^4,10^) to sorb PFAS molecules (>1.5 nm^8^). Amendment with 1% sludge biochar or (activated) high-T wood biochar reduced the level of leaching of perfluorooctanesulfonate (PFOS) from contaminated soil by up to 92–99%,^8,10^ with notably better effectiveness for long-chain than for short-chain (C_4–5_) PFAS (40–70%^8^).

Roughly 5 t of dry weight (dw) (ha of grass)^−1^ year^–1^, approximately one-third of the total harvest, could be turned into 1 t of biochar to be applied on 1 ha per year. Acquiring enough biochar to amend the top 20 cm of a soil (ρ = 1.3 g cm^–3^) with 1% biochar would then take ∼25 years. Using co-pyrolysis with alternative feedstocks such as manure,^11^ crop residues, biosolids,^7,8^ or reeds^10^ could shorten this time frame. Assuming a biochar price of € 1000 t^–1^, the cost would be € 25 000 ha^–1^ plus the cost of the incorporation into the soil plus the cost of fodder yield losses. The overall cost would be lower than that of more intrusive methods^2^ and could further be reduced by incorporating carbon credits of up to € 150 (t of CO_2_)^−1^ by 2030.^11,12^

## Optimizing PFAS Phytoremediation

The effectiveness of PFAS phytoremediation strongly depends on the local conditions and the bioaccumulation factors (BAFs) of the PFAS in the particular soil–plant system. The BAF ranges from ∼10 for short-chain PFBS and PFBA to ∼1 for long-chain PFOS and PFOA.^13^ Phytoremediation times with 5 t of dw plant harvest ha^–1^ year^–1^ are on the order of 50–500 years, underscoring the need to identify hyperaccumulator crops with high BAFs. Such crops will reduce the phytoremediation time for short-chain PFAS to below a few dozen years,^13^ on the same order of magnitude as the time needed to harvest enough biomass to administer 1% biochar.

## Biochar-Amended Fodder to Reduce the Levels of PFAS in Meat and Milk

Biochar administration may improve animal health as well as meat and milk production.^12^ Ruminants have been fed approximately 100–400 g of biochar day^–1^ while consuming 10 kg of dw grass day^–1^.^12^ Biochar reduces PFAS bioaccessibility and thus uptake in the digestive tract, resulting in a reduced level of accumulation in body tissues, reducing chronic animal health risk as well as PFAS levels in milk and meat. Biochar–water distribution ratios, *K*_d_, reach 10^6^ L kg^–1^ for PFOS,^8^ far above grass–water *K*_d_’s (20–50 L kg^–1^).^13^ Thus, biochar could reduce the PFOS bioavailability in the digestive tract by ≤700-fold. Actual reductions may be less due to (i) incomplete fodder–biochar mixing in the rumen and intestine, (ii) natural organic matter reducing the biochar *K*_d_,^8^ (iii) weaker sorption of short-chain PFAS to biochar,^8^ (iv) 250 g of biochar day^–1^ being too little to “depurate” PFAS from a 500 kg ruminant,^14,15^ and (v) digestive fluids increasing PFAS chemical activity.^14^ Conversely, the slightly acidic rumen environment (pH 5.8) could weaken the electrostatic repulsion between the biochar and the PFAS polar headgroups.^4^ Also, digested biochar present in manure could play a role in further sorbing PFAS as well as increasing soil fertility.^12^

## Restoration of PFAS-Contaminated Farmland

Pyrolyzing the entire harvest should be considered a last resort for farmland too contaminated for crop and fodder production. Alternatively, converting only 10–20% of the harvested biomass into biochar could reduce PFAS availability more gradually, offering a long-term solution with climate co-benefits while not compromising farmer income, especially with compensation payments.^16^

There are indications that biochar amendments could be effective over increased time scales. The matrix itself is >80% stable for millennia,^9^ and the sorption strength can increase with time due to slow diffusion into deeper narrow biochar pores^8^ and incorporation into soil aggregates.^17^

The best solution for preventing PFAS contamination of farmland is to prevent it ever entering; however, for already compromised land, application of a phytoremediation–pyrolysis–biochar virtuous cycle could help restore soil quality. Optimization should be done by long-term field trials, including various herbage species and agroforestry approaches and varying pyrolysis conditions. Hyperaccumulators could be grown on 10–20% of the land, pyrolyzed and back-applied, after which grass would be reseeded. Remediation of the entire land would then be achieved after a decade.
